# In vitro evaluation of filling material removal and apical debris extrusion after retreatment using Reciproc blue, Hyflex EDM and ProTaper retreatment files

**DOI:** 10.1186/s12903-023-03579-7

**Published:** 2023-11-21

**Authors:** Passent Abdelnaby, Mohamed Ibrahim, Rania ElBackly

**Affiliations:** 1 Conservative Dentistry Department, MS Student, Endodontics, Faculty of Dentistry, Alexandria University, Alexandria, Egypt; 2 Conservative Dentistry Department, Endodontics, Faculty of Dentistry, Alexandria University, Alexandria, Egypt; 3 Tissue Engineering Laboratories, Faculty of Dentistry, Alexandria University, Alexandria, Egypt

**Keywords:** Apical extrusion, Remaining filling material, Reciproc blue, Hyflex EDM, ProTaper universal retreatment system, Cone-beam computed tomography

## Abstract

**Objectives:**

To evaluate the amount of remaining filing material and apical debris extrusion after retreatment using Reciproc Blue, Hyflex EDM and ProTaper Retreatment Files.

**Materials and methods:**

Thirty-six extracted permanent mandibular first molars with moderately curved mesial roots were selected. Mesiobuccal canals were prepared using the ProTaper Next system up to size X2 and filled using gutta-percha and Adseal sealer via cold lateral compaction. Teeth were randomly divided into three equal groups (*n* = 12): Group 1: Reciproc Blue (RB)(VDW, Germany), Group 2: Hyflex EDM (HEDM)(Coltene/Whaledent, Switzerland), Group 3: ProTaper Universal Retreatment file system + ProTaper Next file system (PTUR + PTN)( Dentsply Maillefer, Switzerland). Eppendorf tubes were used to collect the apically extruded debris. Cone-beam computed tomographic scans were taken prior to and after retreatment and the volume of remaining filling material was assessed at the coronal, middle and apical levels. Statistical analysis was performed using the Kruskal–Wallis test, Friedman's test and Wilcoxon Sign Rank test. Significance level was set at *p* value 0.05.

**Results:**

There were no statistically significant differences among the three groups in the reduction of the volume of the filling material or in the amount of apically extruded debris.

**Conclusion:**

All the tested filing systems showed similar efficacy in removing the filling material, however, none of them could achieve its complete removal. Apical extrusion of debris occurred with all the systems used with no significant difference between the three groups.

## Introduction

The foremost priority during retreatment is to decrease the bacterial load to a level below that required to initiate or maintain a post treatment periapical disease [1].This is achieved by removing the old contaminated filling material, thus enabling irrigating solutions and instruments to access areas of the root canal system where bacterial contamination and pulp remnants may persist.

Different methods have been proposed to remove the old obturation material (gutta-percha and root canal sealer), such as hand files, nickel-titanium (Ni–Ti) rotary and reciprocating instruments with or without solvents and/or ultrasonic inserts [2–4]. However, none of the available techniques was capable of completely removing the filling material as reported by several studies [3, 5].

Various complications may arise throughout the retreatment procedure. Apical extrusion of debris and irrigants is an example of such complications which can be clinically significant as it may trigger an inflammatory reaction [6, 7] with subsequent post operative pain, flare ups, or even delayed or impaired the healing process [8, 9]. Numerous publications have shown that apical extrusion is an unavoidable event during endodontic retreatment therefore, it has been a repeated outcome targeted by recent research [10, 11].

Certain rotary file systems have been designed exclusively for retreatment and improving gutta-percha removal. However, their performance was not reported to be superior to conventional rotary files [12]. A recent systematic review by Rossi-Fedele et al. (2017) [13] reported that conventional files -whether reciprocating or rotary- performed similarly to those files designed specifically for retreatment.

Reciproc Blue (RB) (VDW, Munich, Germany) is a single reciprocating file system that shares the same design features as the M-wire Reciproc with an S-shaped horizontal cross-section,2 cutting edges, and inactive tip [14]. Only the metallurgic properties are altered by the proprietary heat treatment resulting in a visible blue titanium oxide layer on the surface of the file. The Reciproc Blue files are available in similar sizes as the original Reciproc namely: R25, R40, and R50. As per the manufacturer instructions, the R25 file can be used for retreatment. It has a #25 tip size with a continuous taper along the first apical 3 mm of the working part (8%) followed by a decreasing taper up to the shaft [15].

HyFlex EDM (HEDM) (Coltene/Whaledent, Alstatten, Switzerland) was introduced to shape the root canals in continuous rotation using a single-file technique. It is manufactured via an electrical discharge machining process (EDM), which is a noncontact machining procedure that permits accurate material removal using pulsed electrical discharge. As per the manufacturer, this machining process should harden the surface resulting in superior cutting efficacy and fracture resistance [16]. HEDM “one file” has a #25 tip size with a constant 0.08 taper in the apical 4 mm of the instruments that decreases progressively up to 0.04 in the coronal part of the instrument [17].

The ProTaper Universal retreatment system (PTUR) is a rotary system especially designed for retreatment. It consists of 3 instruments that are used in continuous rotation and have a convex triangular cross section: D1 (tip 30 and taper 0.09) has an active tip to help the initial penetration of filling material at the coronal third, D2 (tip 25 and taper 0.08) used at the middle third, and D3 (tip 20 and taper 0.07) used till the full working length [18].

Although RB and HEDM files were not specifically designed for retreatment cases, their superior cutting efficiency as proved by various studies, combined with their file design and controlled memory effect [16] may make them a valid choice during filling material removal [19, 20].

Although some recent studies had evaluated apical extrusion of debris after using RB and HEDM during initial root canal treatment, no study was found to compare debris extrusion during retreatment using these two file systems. Thus, to the best of the authors' knowledge, the present study is the first in vitro study to compare the performance of the RB and the HEDM files during retreatment in regard to the following two parameters simultaneously: filling material removal ability and apical extrusion of debris.

Therefore, the null hypothesis of this study was that there would be no significant differences between the three tested file systems (RB, HEDM and PTUR + PTN) with respect to filling material removal ability and the amount of apically extruded debris.

## Materials and methods

### Sample selection

This research was approved by the ethics committee of the Faculty of Dentistry, Alexandria University. IRB NO: 00010556 – IORG 0008839on (13-9-2020).

Thirty-six extracted permanent mandibular first molars that were extracted due to periodontal or orthodontic reasons were collected from the out-patient clinic of the Oral Surgery Department, Faculty of Dentistry, AlexandriaUniversity. Collected teeth were cleaned from debris, calculus and organic tissues, then immersed in 5.25% NaOCL (Clorox Co, 10 th of Ramadan, Egypt) for 10 min and stored in 0.9% saline solution (El Fath For Drug and Cosmetics Industry (FIPCO), Borg El Arab, Egypt)until use to avoid dehydration. Digital periapical radiographs were taken for sample selection. Teeth with root caries, open apices, resorptive defects, calcifications, cracks, and previous root canal treatment were excluded [2, 10]. Only mesial roots having two separate mesial canals and independent apical foramina.(Vertucci class IV) [21], mesiobuccal canals having an initial apical diameter not greater than a size # 10 K-file, and a curvature degree ranging between 10^0^ and 20^0^ were included in this in vitro study. The curvature angles were measured according to Schneider’s technique [22] using the imageJ software (National Institutes of Health, Bethesda, MD).

### Sample size calculation

Sample size was estimated based on assuming 5% alpha error and 80% study power. The mean ± SD of the apically extruded debris weight in ProTaper retreatment files system was 0.39 ± 0.21 mg, [23]while, it was 1.42 ± 0.4491 mg [24] and 1.177 ± 1.6248 mg [10] in Reciproc Blue and Hyflex EDM, respectively. Using F test with pooled SD of 0.7613, sample size was calculated to be 12 specimens per group. Sample size was based on Rosner’s method [25] calculated by Gpower 3.0.10 [26].

### Cleaning and shaping

A conventional access cavity was prepared in each tooth using a high speed round bur and an endo—Z bur (DentsplyMaillefer, Ballaigues, Switzerland) under copious water cooling. The crowns of all teeth were sectioned using a diamond stone to obtain a standardized tooth length of 15 ± 1 mm. A size 10 k-file (DentsplySirona,Tulsa, USA) was introduced into the mesiobuccal canal of each tooth until the tip became just visible at the apical foramen to confirm canal patency. The working length was then established at 1 mm short from the apical foramen. A glide path was established using size 10 and 15 k-files (DentsplySirona,Tulsa,USA). ProTaper Next (PTN) system (DentsplyMaillefer, Ballaigues, Switzerland) activated by the X-Smart plus endodontic motor (DentsplyMaillefer, Ballaigues, Switzerland) was used for root canal preparation according to the manufacturer's instructions. PTN X1 file (17/04 apical taper) was introduced into the canal until reaching the working length. Then the PTN X2 file (25/06 apical taper) was used to finish canal preparation. During instrumentation, canal irrigation was done after each file change with 2 ml of 2.5% sodium hypochlorite (Clorox Co, 10 ^th^ of Ramadan, Egypt) and patency was re-assessed using a size #10 k –file. Irrigation was performed using a 30 gauge side vented needle (Endo-Top®, PPH CERKAMED, StalowaWola, Poland) with an insertion depth of 2 mm short of the working length (WL). After completion of instrumentation, the smear layer was removed with the final rinse of 5 ml of 17% EDTA(Calix E, Dharma, Miami, Florida, USA) for one minute followed by 5 ml of 2.5% NaOCl.

### Root canal filling procedures

The root canals were dried using paper points and canal obturation was done using the cold lateral compaction technique. In brief, a master X2 gutta-percha cone (DentsplySirona,Tulsa, OK) was tried in each canal for length and fitness. Afterwards, the X2 gutta-percha cone was coated by Adseal resin based sealer (META BIOMED CO,.LTD, Korea) and placed up to the working length. Then, lateral compaction of accessory cones (size 20, 0.02 taper) (META BIOMED CO,.LTD, Korea) was done until the canal was filled. A heated hand plugger was used to remove the excess gutta-percha at the level of the canal orifice. The canal orifices were sealed using a small piece of cotton and temporary filling material (MD-Temp;META BIOMED CO,.LTD, Korea). The distal root was removed using a diamond disc mounted on a straight hand piece, to allow radiographic evaluation of the obturation in the mesiobuccal canal without superimposition [2]. Digital periapical radiographs were taken in both the bucco-lingual and mesio-distal directions to verify the quality of the root filling. Samples showing voids, underfilling or overfilling were discarded and new samples were prepared. Teeth were stored at 37 °C in 100% humidity for two weeks to allow the sealer to set [18].

## Grouping

The specimens were numbered and randomly allocated to three equal groups (*n* = 12) according to the system used for retreatment. Randomization was done using permuted block technique. The randomization scheme was generated by using (Random allocation) software [27].

- Group 1: Reciproc Blue group.
- Group 2: Hyflex EDM group.
- Group 3: ProTaper Universal Retreatment file system followed by ProTaper Next X2file.

### Cone beam computed tomography (CBCT) scanning

Teeth were mounted into a wax platform (4 in each block) and precisely labeled before cone beam computed tomography scanning. A J. Morita veraview × 800 cone beam 3D imaging system (JMORITA MFG. CORP. Kyoto Japan) was used to scan the specimens at two stages: immediately after obturation and after retreatment. All specimens were scanned in the same position with the same parameters: 90 kV,8 mA, 0.08 mm isometric voxel size, and a field of view (FOV) of 80 mm x H 40 mm. After the acquisition of the CBCT images, the DICOM files were imported into an image analysis software program "Mimics"(Materialise, Belgium).

### Root canal retreatment

#### Group 1: Reciproc blue group

Reciproc Blue R25 (25/ 0.8) instrument was activated by the X-Smart plus electric motor applied in a reciprocating motion.The file was introduced into the canal in a slow in-and-out pecking motion with a 3-mm amplitude limit. A gentle apical pressure combined with a lateral brushing motion against the canal walls was applied. The instrument was removed from the canal after 3 pecking movements to be cleaned. The Reciproc Blue R25 instrument was used to remove the root fillings until the working length was reached.

#### Group2: Hyflex EDM file

Hyflex EDM size (25/0.08) was used up to the working length. The file was activated by the X-Smart plus electric motor at a speed of 500 rpm rotary and a torque of 2.5 Ncm [10]. Hyflex EDM and Reciproc Blue files were used only once as per the manufacturer's recommendations.

#### Group 3: ProTaper Universal Retreatment (PTUR) files group + ProTaper Next (PTN)

The PTUR system was used at speed 500 rpm and torque 2Ncm [12]. The D1 file was used to remove the canal filling material at the coronal third of the canal, while the D2 and D3 (tip 20 and taper 0.07) were used for the middle and apical thirds,respectively. The PTN X2 file (25/06) was then used up to the working length at speed 300 rpm and torque 4Ncm. Each instrument of the PTUR system was used for 3 canals only [28] while the PTN X2 files were used only once.

In all groups, no solvent was used during filling material removal. Throughout the retreatment procedure, a total of 2 ml of 2.5% NaOCl solution was delivered for each tooth using a 30 gauge side vented needle with an insertion depth of 2 mm short of the working length. At the end of the procedure, patency was confirmed using a #10 K file. Retreatment was deemed complete in all groups when the working length was reached, the canals were smooth and no debris of gutta-percha or sealer was visible on the instrument surfaces. In addition, periapical radiographs were used to confirm the complete removal of filing material. The irrigation, cleaning and shaping procedures were completed by one operator to exclude any variation and to eliminate bias. Instrument fractures during retreatment were recorded.

### Assessment of apical extrusion

Assessment of debris extrusion was done using a modified version of the experimental model reported by Myers & Montgomery (1991) [29]. Empty eppendorf tubes were used to collect debris and irrigation from each tooth. First, tubes were numbered and weighed without the stopper using a microbalance (Radwag, Random, Poland) with 10^–4^ g sensitivity. The measurement for each tube was performed three times, and the average of the measurements was calculated and referred to as (W1). A round hole was created in the stopper of each tube to fit the mesial root which was inserted up to its cemento-enamel junction and fixed with cyanoacrylate to prevent irrigant leakage. A 27-gauge bent needle (Genject,Ankara,Turkey) was inserted into the stopper to balance the internal and external air pressures. The stopper containing the tooth and the needle was re- attached to the eppendorf tube which was fitted then into a glass flask to avoid any grease or debris from fingerprints onto the tubes and to provide better stability during retreatment. The flask was covered with foil to prevent the operator from observing the root apex during root canal instrumentation. After retreatment, the stopper with the mesial root fixed to it were removed from the tube and the external surface of the apical third was rinsed with 1 mL distilled water to release and collect any debris that could have extruded and remained attached to the external surface at the apical foramen.The eppendorf tubes were incubated at 37 °C for 14°days [10] to allow all liquid to dry out before a second weighing (W2) was performed using the same microbalance. All the pre-and post-weights of the tubes were obtained in triplicates to obtain the mean weight for each specimen. The total amount of apically extruded debris was then calculated as the difference between the pre- and post weights (W1-W2).

### Analysis of remaining root canal filling material

In order to calculate the filling material volume; image segmentation by thresholding was first performed in the sagittal view to distinguish the filling material from the tooth structure.

### Manual thresholding and three dimensional volumetric image analysis

First, a threshold value at which the representative pixels of dentin were excluded was determined in the sagittal view using the uninstrumented/sound mesio lingual half of the tooth as a reference. This was done by gradually increasing the minimum threshold value on the scan histogram until the dentinal walls of all the samples in all slices were not highlighted anymore (Fig. 2).This method was repeated for each CBCT data set individually and the values were recorded. Secondly, the mean value -obtained from the recorded values- was calculated (4032 ± 91.1) and used as a pre-determined threshold value during the volumetric image analysis to standardize the method of assessment for all the scans i.e. values above this mean threshold value were used to determine the segmentation mask which was later imported to measure the filling material volume (Fig. 2).

### Volume acquisition

**Fig. 1:**
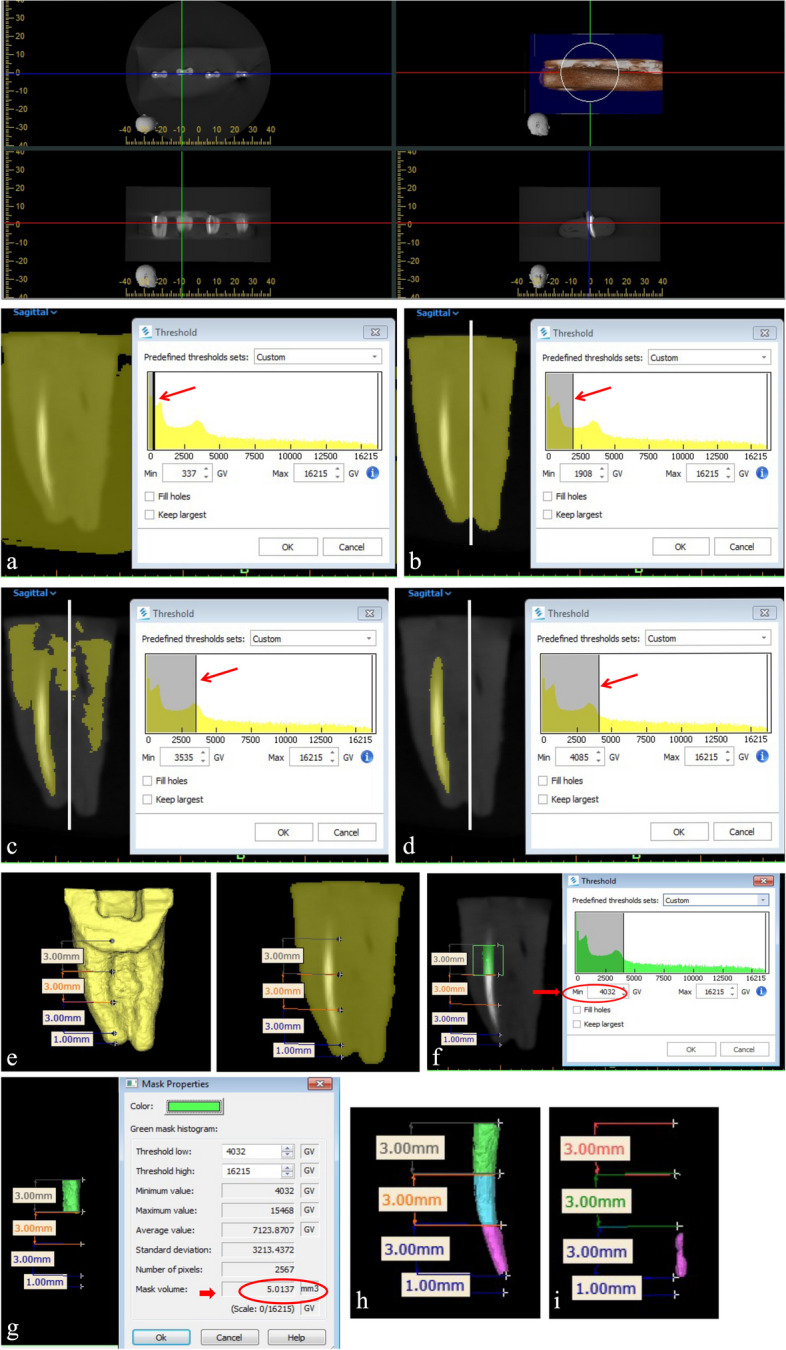
Manual thresholding technique (**A**-**D**) and Volume acquisition technique (**E**-**I**). Manual thresholding technique; (**A**-**D**) represent the sequential process of manual thresholding to select the segmentation mask. Arrow indicates manual threshold selection on the scan histogram while the white vertical line represents the virtual distinction between the two halves of the root. Volume acquisition technique(**E**-**I**); (**E**) the mesial root was divided into three thirds: apical, middle, and coronal after leaving 1mm from the apex; (**F**) The standardized pre-calculated threshold value was imported to identify the filling material at the coronal third; (**G**) The software calculated the volume of the segmented filling material in (mm^3^); (**H**) the total volume of the filling material before retreatment; (**I**) the total volume of the filling material after retreatment

**Fig. 2:**
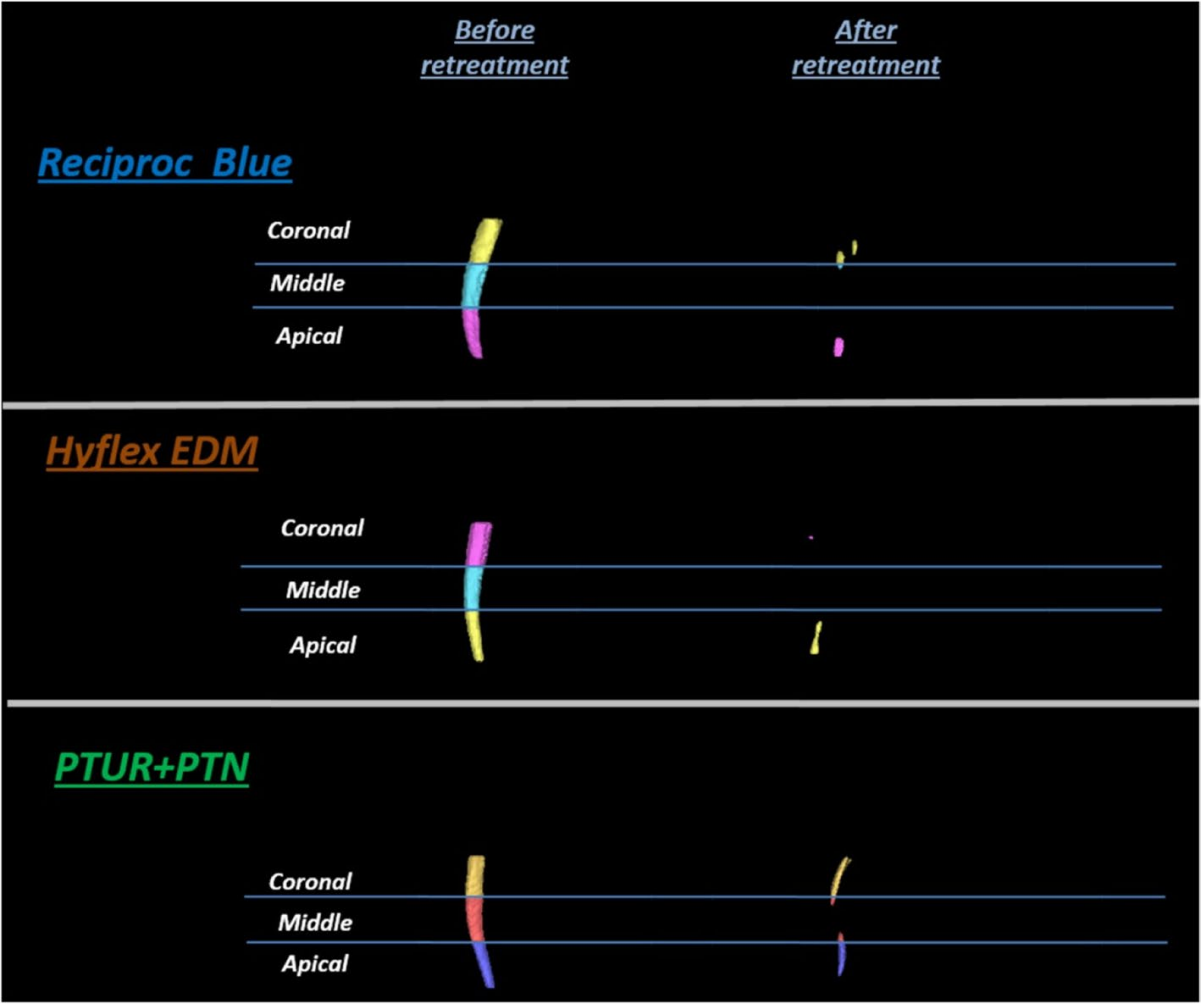
Reconstructed three-dimensional CBCT images representing the filling material before and after retreatment in representative samples from the three study groups:RB,HEDM and PTUR + PTN

To obtain the pre operative and post operative filling material volume in mm^3^, the mesial root was divided into three thirds (3 mm each): apical, middle, and coronal after leaving 1 mm from the apex (Fig. 2). A mask was then cropped using the sagittal view, for the third of interest (Fig. 1). Afterwards, the standardized pre-calculated threshold value was imported to identify the filling material. Manual refinements were done using the "edit mask " tool to erase highlighted volumes that were away from the region of interest such as sealer in the ismuth region or apical ramifications.The software then calculated the volume of the segmented filling material in (mm^3^) (Fig. 2).The total volume of the filling material in a canal was calculated by the addition of the volumes of the three thirds. The percent reduction of the volume of the filling material on canal walls was calculated with the following equation: [(volume before retreatment–volume after retreatment)/ values before) × 100]. No attempts were made to differentiate between the gutta-percha and sealer.

### Inter and intra- examiner reliability

After a calibration session, two experts in both quantitative and qualitative CBCT image analysis performed image thresholding and volume acquisition while being blinded to which group the samples belonged. Using the thresholding value obtained by each examiner, the filling material volumes were calculated for each specimen and recorded.

### Statistical analysis

Normality was checked using Shapiro Wilk test, Boxplot and descriptives. Non- parametric tests were adopted. Comparisons between the three study groups were done using Kruskal Wallis test. Comparisons between different regions (coronal, middle and apical) within each group were done using Friedman's test. Wilcoxon Sign Rank test was used to compare between the volume of the filling material before and after retreatment within each group. Significance level was set at *p* value 0.05. Data was analyzed using IBM SPSS version 23.

## Results

Intra and inter examiner agreement was assessed. Intraclass Correlation Coefficient (ICC) [30] was calculated to be 0.95–0.99 indicating excellent reliability. Thus, determining the threshold value using the previously described method yielded reliable filling material volume measurements.

### Residual filling material

**Fig. 3:**
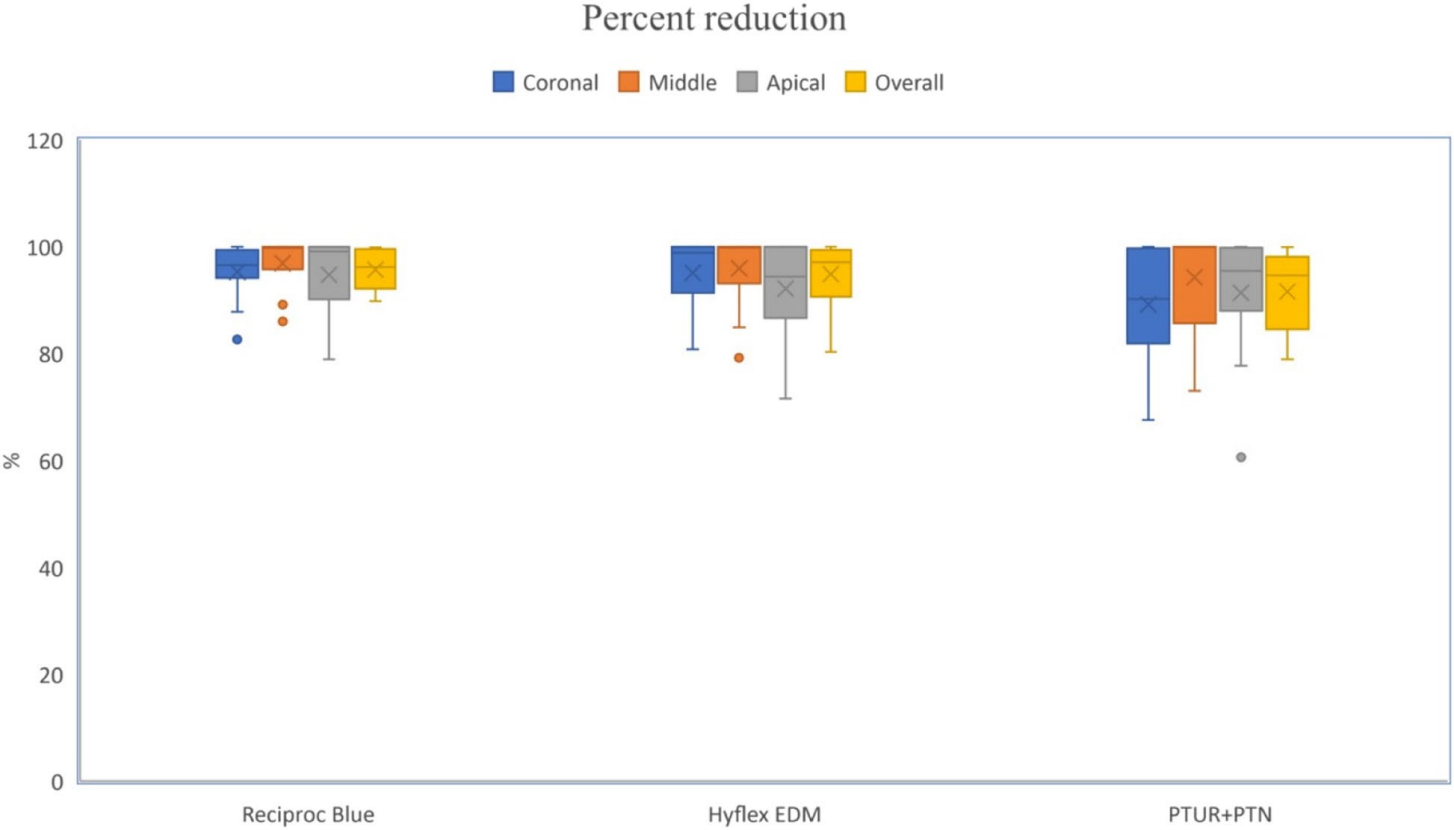
A boxplot showing the mean, median, minimum and maximum values of the percent reduction for all the tested groups

The results of the current study showed a significant reduction in the volume of filling material after the use of the three file systems indicating their efficiency/efficacy during retreatment (*P* < 0.05) (Table 1). The intergroup comparisons showed no significant difference between groups in the volume of the filling material before retreatment -considering the overall analysis and the analysis of each third individually – allowing for reliable intergroup comparison (Table 1). No statistically significant difference was observed between the three groups during retreatment with respect to their filling removal ability either in total (entire root canal) or considering each third individually. However, it was shown that the Reciproc Blue removed the highest amount of filling material at all the studied thirds as well as at the entire canal length, followed by the Hyflex EDM and finally the PTUR + PTN (Fig. 3 and Table 2).

**Table 1:** Filling material volume (mm^3^) at different regions before and after retreatment with the different instruments. Given are the means with standard deviation (SD) and the medians with the interquartile ranges

Coronal third					
		**Reciproc Blue**	**Hyflex EDM**	**PTUR + PTN**	***P* value**
Before retreatment	Mean (SD)	5.23 (1.04)	4.79 (0.88)	5.33 (0.85)	0.228
	Median (IQR)	5.30 (1.41)	4.85 (1.51)	5.55 (1.00)	
After retreatment	Mean (SD)	0.25 (0.26)	0.24 (0.37)	0.57 (0.54)	0.215
	Median (IQR)	0.18 (0.31)	0.07 (0.43)	0.55 (1.01)	
**P value**		0.002*	0.002*	0.002*	
**Middle Third**					
		**Reciproc Blue**	**Hyflex EDM**	**PTUR + PTN**	**P value**
Before retreatment	Mean (SD)	3.86 (0.55)	3.72 (0.89)	4.24 (0.75)	0.144
	Median (IQR)	3.80 (0.70)	3.30 (1.27)	4.15 (0.80)	
After retreatment	Mean (SD)	0.12 (0.19)	0.20 (0.37)	0.26 (0.43)	0.998
	Median (IQR)	0.01 (0.17)	0.01 (0.31)	0.00 (0.62)	
***P* value**		0.002*	0.002*	0.002*	
**Apical third**					
		**Reciproc Blue**	**Hyflex EDM**	**PTUR + PTN**	**P value**
Before retreatment	Mean (SD)	2.39 (0.41)	2.28 (0.48)	2.61 (0.66)	0.391
	Median (IQR)	2.25 (0.46)	2.15 (0.73)	2.75 (1.03)	
After retreatment	Mean (SD)	0.13 (0.18)	0.18 (0.20)	0.22 (0.27)	0.718
	Median (IQR)	0.02 (0.22)	0.15 (0.36)	0.13 (0.33)	
***P* value**		0.002*	0.002*	0.002*	
**Total**					
		**Reciproc Blue**	**Hyflex EDM**	**PTUR + PTN**	**P value**
Before retreatment	Mean (SD)	11.49 (1.79)	10.78 (2.13)	12.17 (1.86)	0.197
	Median (IQR)	10.95 (2.05)	10.28 (3.53)	12.25 (3.33)	
After retreatment	Mean (SD)	0.49 (0.39)	0.62 (0.82)	1.04 (0.99)	0.393
	Median (IQR)	0.49 (0.84)	0.24 (0.88)	0.69 (1.71)	
***P* value**		0.002*	0.002*	0.002*	

^*^Statistically significant at *p* value ≤ 0.05

**Table 2:** Percent reduction of the filling material volume at different regions after preparation with the different instruments.Given are the means with standard deviation (SD) and the medians with the interquartile ranges

			**Reciproc Blue**	**Hyflex EDM**	**PTUR + PTN**	***P* value**
Percent reduction (%)	Coronal	Mean (SD)	**95.29** (5.21)	**95.13** (6.98)	**89.21** (10.85)	0.258
		Median (IQR)	96.57 (5.26)	98.85 (8.62)	90.26 (17.83)	
	Middle	Mean (SD)	**96.96** (4.71)	**96.01** (7.03)	**94.28** (9.63)	0.997
		Median (IQR)	99.76 (4.21)	99.84 (6.85)	99.96 (14.29)	
	Apical	Mean (SD)	**94.76** (7.15)	**92.16** (9.03)	**91.38** (11.81)	0.714
		Median (IQR)	99.09 (9.84)	94.39 (13.32)	95.46 (11.83)	
	*P* value		0.856	0.975	0.078	
	Total	Mean (SD)	**95.74** (3.58)	**94.88** (5.93)	**91.62** (7.75)	0.502
		Median (IQR)	96.20 (7.39)	97.14 (8.79)	94.63 (13.60)	

Considering the entire root canal, the mean percent reduction of the filling material was 95.74%; Reciproc Blue, 94.88%; Hyflex EDM and 91.62%; PTUR + PTN. While in the coronal third, the mean percent reduction of the filling material was 95.29%; Reciproc Blue, 95.13%; Hyflex EDM and 89.21%; PTUR + PTN. Concurrently, in the middle third, the mean percent reduction was 96.96% with the Reciproc Blue, 96.01% with the Hyflex EDM and 94.28% with the PTUR + PTN, whereas, in the apical third it was 94.76% with the Reciproc Blue, 92.16% with the Hyflex EDM and 91.38% with the PTUR + PTN.

The intragroup comparisons, in all groups, showed no statistically significant difference between thirds in regard to the reduction of the filling material volume.( Fig. 3 and Table 2).However, the middle third showed the least amount of filling material in all groups while the highest amount of residual filling material was found at the apical third with RB and HEDM and at the coronal third with PTUR + PTN.

No procedural errors were recorded in the ProTaper Universal Retreatment system + ProTaper Next and the Reciproc Blue groups. However, one file fractured during retreatment in the Hyflex EDM group and this sample was replaced with a new one.

### Apical extrusion

**Fig. 4:**
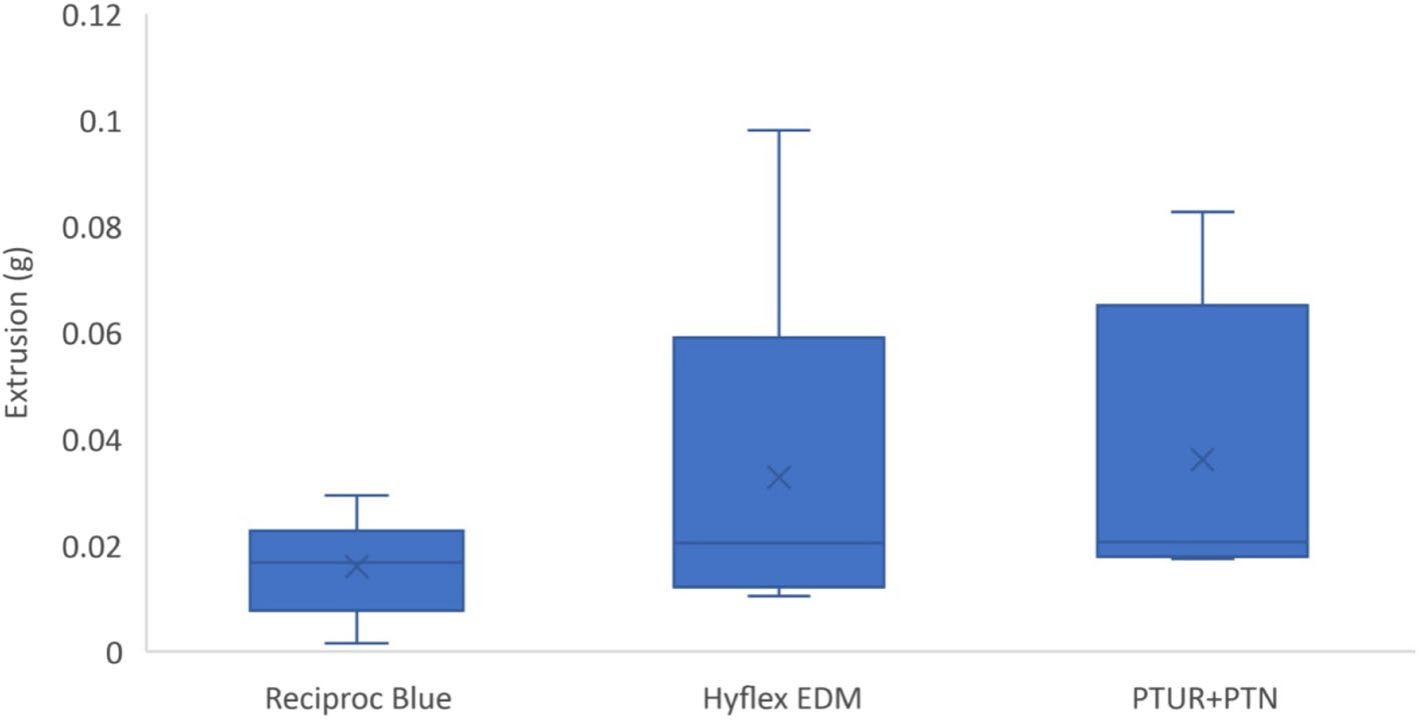
A boxplot showing the mean, median,minimum and maximum values of the weight of the extruded debris (g) for all the tested groups

The results showed that apical extrusion of debris occurred in all groups regardless of the file system used.( Fig. 4 and Table 3) There were no statistically significant differences in the weight of extruded debris between the three groups at a P value > 0.05. However, the PTUR + PTN showed the highest amount of extruded debris compared to the other two groups as the mean weight of debris in grams was 0.04.While the Hyflex EDM group showed a mean weight of 0.03 g followed by the Reciproc Blue group with a mean weight of 0.02 g.

**Table 3:** Amount of apically extruded debris (g) after preparation with the different instruments. Given are the means with standard deviation (SD) and the medians with the interquartile ranges

		**Reciproc Blue**	**Hyflex EDM**	**PTUR + PTN**	***P* value**
Weight of Extruded debris (g)	Mean (SD)	**0.02** (0.01)	**0.03** (0.03)	**0.04** (0.03)	0.080
	Median (IQR)	0.02 (0.02)	0.02 (0.05)	0.02 (0.05)	

## Discussion

This study evaluated the efficacy of a reciprocating single-file system (RB), a rotary single-file system (HEDM) and a rotary multi-file system (PTUR + PTN) in endodontic retreatment of moderately curved root canals in regards to the amount of apically extruded debris and remaining filling material after retreatment.

Although there is no clinical evidence on how the amount of remaining filling material and apically extruded debris would affect the retreatment and healing outcome,it is reasonable to assume that increased amounts of residual filling material, especially at the apical region, may increase the probability of sheltering bacteria, resulting in persistent apical periodontitis and compromised retreatment [31–33]. Whereas, it would be plausible to suppose that the increased amounts of extruded debris may trigger a more severe inflammatory response [6, 9, 34], with subsequent pain, flare-ups, and in the most severe cases, delayed or impaired healing as assumed by some authors [8, 9, 35]. This may be attributed to the findings of Siquiera [8] and Caviedes et. al [6] who reported that the intensity of inflammation increases with the intensity of the insult and the extent of tissue damage.

Mandibular molars were selected in this study as the incidence of flare-ups was shown to be significantly higher in mandibular molars [36]. Thus exploring apical extrusion -which is considered one of the principal causes of postoperative pain and flare-ups- [8] using mandibular molars is relevant from a clinical standpoint.

During retreatment, several factors may affect the amount of extruded debris as well as the remaining filling material such as: the features of the file used ( design, number of files, taper, tip size, alloy treatment,cross-section,cutting efficiency and motion kinematics) [10, 18, 37–39], and the internal anatomy of the canal (canal volume, degree of curvature, length, canal format, configuration, and apical size) [10, 20, 40–42].

In the present study, anatomic standardization was achieved as much as possible to reduce anatomy-related biases and to ensure comparability of the groups creating a reliable baseline. Standardization of initial apical diameter, angle of curvature, length of samples, canal configuration and the initial volume of the filling material before retreatment was established. Also, standardization of the apical limit and final apical preparation size (# 25) during retreatment was done, allowing better comparison between the groups,as these may be variables that may affect the amount of apical extrusion of NaOCL and debris [6, 43, 44]. Additionally, the generally accepted Myers and Montgomery method [29] was used as it is a repeatable approach and thus allows for further meta-analysis between the different studies.

Many in vitro debris extrusion studies have used distilled water for irrigation instead of sodium hypochlorite to avoid sodium crystallization as reported by previous authors [2, 10, 37] whereas others used sodium hypochlorite [14, 43, 45, 46]. In the present study, sodium hypochlorite (NaOCL) was used as the irrigating solution during retreatment rather than distilled water to mimic the clinical condition as reported by Nevares et al. [2, 18] and to study not only the physical aspect of the irrigant but also the biological one since sodium hypochlorite can dissolve organic debris and dentin which may increase the extrusion of debris when compared to distilled water as reported by Ozlek et al. [46]. NaOCL has a higher density and specific gravity than water which may have an impact on the amount of extruded debris as well. Also, the crystals formed after the evaporation of NaOCL should not be neglected and should be taken into consideration since they may act as a representative of the volume of the extruded irrigant [43, 47].

It is worth mentioning that the volume [48], concentration, form (liquid or gel) of the irrigant used during retreatment [46, 49] in addition to the type and penetration depth of the needle were standardized for all specimens to avoid any confounding factors since these factors may affect the amount of apically extruded debris as reported by previous studies [43, 46, 48, 49].

No solvent was used during retreatment in this study following the protocol of previous studies [3, 14]. This was mainly to eliminate a possible confounding factor and to avoid the chemical plastification of gutta-percha which may result in the adherence of a fine layer on the canal walls [3], as well as pushing this soft gutta-percha into complex canal anatomies making the cleaning process difficult [14].

Regarding the evaluation of the residual filling material, various methods have been described: longitudinal sectioning of the roots [50], radiographic analysis [51] and clearing techniques [52]. However, CBCT is considered a more preferable method as it is nondestructive and provides a 3D analysis of filling material volume (in mm^3^) which is more precise than the surface area measurement obtained with the other methods [53].

Although, CBCT has some drawbacks such as poor contrast, noise and artifacts such as the beam hardening effects that may be caused by the radio opaque filling material, [54], this can be neglected with proper machine settings and parameters. In the present study, a 0.08 mm isometric voxel size was used to reconstruct the images. Such a small voxel size minimizes the presence of artifacts and enhances the image quality [55]. Micro-CT analysis has also been used for volumetric image analysis [5]. Ideally, micro-CT has better resolution compared to CBCT and shows more accurate results regarding volume estimation. However, a study by Yilmaz et al. [53] reported that the residual filling material volume measurements obtained from CBCT images highly correlated with those from the micro-CT suggesting that CBCT images have a great potential to be used for volumetric analysis of remaining root canal filling material after retreatment.

Manual thresholding was used for image segmentation. While an advantage of this method is that it is simple based on the assumption that images are formed from regions with different grey levels [56], a drawback of this method is that it is observer-dependent. This makes the segmentation process prone to intra and inter-examiner variability due to the difficulty of visualizing definite boundaries separating the filling material and the dentin in the filled part of the root. To overcome the aforementioned problem, the unfilled mesiolingual part of the root was used as a reference (the control) during the exclusion of the of the dentin representative pixels from the whole mask. This method yielded reproducible filling material volume measurements and excellent intra and inter-examiner agreement.

In the present study, all tested instrumentation systems effectively removed the filling material corroborating the results of previous studies approving the retreatment ability of PTUR, RB and the HEDM [10, 14, 18, 19]. However, none of these files rendered the canals completely free from filling material. This is consistent with previous studies which stated that complete elimination of the filling material was not possible or very rare- regardless of the instrumentation technique used- due to the anatomic complexities of the root canal system and the failure of endodontic instruments to reach all areas of the root canal [5, 12, 13, 39, 57].

No statistically significant difference was found between the three groups during retreatment with respect to their filling removal ability either in total (entire root canal) or considering each third individually. These results are consistent with that of previous studies showing no statistically significant difference between rotation and reciprocation during retreatment [5, 13, 18, 57, 58]. For example, two recent studies by Bago et al. [18, 39] concluded that the RB and PTUR performed similarly during retreatment. On the other hand, other authors reported significantly better performance of rotation when compared to reciprocation during retreatment [42, 59]. However, others reported that reciprocating files significantly outperformed rotary files [39, 51]. These conflicting results when comparing different continuous rotation and reciprocating rotary instruments during retreatment are likely due to several factors -apart from kinematics -that may affect the performance of the instrument used; most importantly the special features of the file (such as tip size,cross- sectional design, taper, and alloy treatment**)**, the canal anatomy (oval, straight, or curved), the filling type, the filling technique, the use of solvents, the operator experience, the number of samples in each group and the assessment protocol used [18].

Regarding the apical extrusion of debris, the present study showed that debris extrusion occurred with all systems. This is consistent with previous studies which reported that apical extrusion of debris is an inevitable event during retreatment regardless of the instrumentation technique used [10, 11, 14]. No statistically significant difference was observed between the three studied groups. This was consistent with studies showing no statistically significant difference in the amount of extruded debris between rotation and reciprocation either during retreatment [2, 9–11]or initial root canal treatment [46]. A recent randomized controlled clinical trial by Çanakçi et al. [60] reported no significant difference between the Protaper Universal Retreatment + ProTaper Gold, Hyflex EDM and the Reciproc Blue in the incidence, intensity or duration of postoperative pain after retreatment suggesting that the three file systems may apically extrude similar amounts of debris.

Other studies showed opposite results where reciprocation produced less extruded debris when compared to rotation either during retreatment [34, 61] or initial root canal treatment [37, 62]. On the other hand, several studies have reported that rotary instruments were associated with less debris extrusion compared with reciprocating instruments during retreatment [63, 64].

This controversy between the results is usually attributed to the heterogeneity of research methodologies and the use of different apical extrusion experimental models [65, 66], differently designed instruments,different numbers of files,different irrigant volumes, irrigant delivery systems, different canal anatomies or variability of the dentin microhardness among the samples [6, 11, 67], emphasizing the fact that motion kinematics is one of the factors and not the only factor that needs to be considered upon analyzing the complex etiology of apical extrusion of debris [11, 68].

Despite the fact that no significant difference was observed between the three file systems, in regards to both parameters – debris extrusion and filling material removal– RB showed the best performance followed by the HEDM and finally the PTUR + PTN. This may be attributed to the S-shaped cross section with the 2 sharp cutting edges of the RB file which gives the file the superior cutting efficiency and provides space for coronal displacement of debris, whereas the machining process of the HEDM file may have increased its cutting efficiency together with the different cross sectional design along the shaft which may have led to better coronal debris displacement.

Many studies have reported that increased file taper may cause greater debris production due to the more aggressive cutting as well as the canal straightening inherent to the decreased flexibility caused by the greater tapers [11, 38].On the other hand, increased file taper may enhance the filling removal ability of the file due to increased contact with the canal walls [59, 69].In the current study, increased taper at the last apical 3 mm of both the RB and the HEDM (8%) may have resulted in better filling removal ability when compared to the X2 file(6% taper for the 3 mm at the tip).

Unexpectedly, this increased taper at the last apical 3 mm of both the RB and the HEDM (8%) have generated a lower amount of extruded debris when compared to the Protaper next X2 file's less tapered design; the last file used with the PTUR + PTN group (#25, 6%). Thus, according to the present study, taper did not appear to cause an increase of apical extrusion of debris corroborating previous studies [37, 40, 62, 64].

This finding may be attributed to the controlled memory effect and flexibility inherent to the alloy heat treatment of both the RB and HEDM which might have outweighed the effect of greater tapers. Although the effect of alloy heat treatment was not studied as an independent variable in this study, it can be assumed that alloy heat treatments with the subsequent flexibility and controlled memory effect would result in less amount of apically extruded debris as reported by previous studies [24, 70, 71].

Although it is reasonable to expect that the greater taper at the coronal part of a file would also result in better filling removal coronally, this was not the case in the present study. The increased taper at the coronal part of the X2 file did not enhance the filling removal and the PTUR + PTN group showed a higher amount of residues on the canal walls at the coronal third when compared to that left by the RB and HEDM at the coronal third. This finding was in line with the studies by Gad et al. [58], Serefoglu et al. [11] Monguilhott Crozeta et al. [69] who reported that PTUR group left the highest amount of residual filling coronally when compared to other rotary file systems. Reciproc Blue and Hyflex EDM might have been able to perform a more efficient brushing motion at the coronal part because to their improved flexibility and high cutting power.

Upon intragroup analysis, it was observed that all the studied file systems performed the best- in regard to the remaining filling material- at the middle third when compared to the performance at the coronal and apical thirds with no significant difference between any of the regions. Although the coronal third is more accessible, it is considered the widest part of the canal often making retreatment procedure more difficult. While at the apical area, files may have performed less efficiently because it is a less accessible area making retreatment harder as reported by previous studies [72, 73]. Also,the fact that no apical enlargement was done beyond the apical size of the primary treatment may have contributed to this finding.

A limitation of this study is that obtained results may not be identical in a clinical setting. This might be due to the fact that more complex anatomies such as severely curved or oval root canals may exist clinically posing more challenges [14, 20]. In addition, the presence of the periapical tissue resistance found in vivo is another important reason. Previous studies attempted to simulate the resistance by utilizing agarose gel [74] and a floral sponge [75], or to perform the study in cadavers or in patients by adding contrast medium to the irrigant [76] or measuring the inflammatory markers in the periapical fluid [77]. However, it was found that all those proposed attempts were found to have limitations [74, 78]. Additionally, studies conducted on patients were difficult to standardize due to the presence of multiple host-related and operator-related factors owing to the individual variations that is expected from clinical studies [6, 79]. Thus, in the present study, no attempts were made to simulate the back pressure by the periapical tissues and this condition was standardized among the three groups.Another limitation of this study is that the micro-CT was not used. This may have prevented the distinction between sealer and gutta-percha. Also, this may have limited the use of a simple segmentation process and automatic segmentation tools which are easier, less time consuming and not observer dependent [80].

Despite the aforementioned limitations, an in vitro study may act as a baseline for future clinical studies and in some cases, the only approach to evaluate different aspects such as apical debris extrusion, not to mention that it provides more standardized conditions, enabling reliable intergroup comparisons.

As there was no significant difference between the performances of the three studied file systems during retreatment in regard to the amount of residual filling material and extruded debris, the null hypothesis of this study was accepted.

Future investigations are required to examine canal transportation and remaining dentin thickness following retreatment in moderately and severely curved canals using the same files as in the current study, to gain a more comprehensive understanding of their efficacy during retreatment.

## Conclusion

All the tested file systems showed similar efficacy in removing the filling material. However, none of them could achieve complete removal of filling material from the samples. Apical extrusion of debris occurred with all the systems used with no significant difference between the three groups.

## Data Availability

The datasets used and/or analysed during the current study available from the corresponding author on reasonable request.
